# Redefining virtual mental health services: youths’ perspectives and ideal features

**DOI:** 10.1192/j.eurpsy.2025.480

**Published:** 2025-08-26

**Authors:** E. Samari, J. A. Vaingankar, S. Chang, A. S, Y. C. Chua, C. Tang, Y. P. Lee, M. Subramaniam, S. Verma

**Affiliations:** 1 Research Division; 2 Department of Psychosis; 3 CHAT, Centre of Excellence for Youth Mental Health, Institute of Mental Health; 4 Saw Swee Hock School of Public Health, National University of Singapore; 5 Institute of Mental Health; 6 Duke-NUS Medical School, Singapore, Singapore

## Abstract

**Introduction:**

The COVID-19 pandemic prompted a significant shift in our approach to healthcare, leading to the widespread adoption of virtual healthcare services, including mental healthcare. In this context, understanding and incorporating the unique perspectives of youths is crucial for improving virtual mental health services for this population.

**Objectives:**

This qualitative study explores the ideal features of virtual mental health services among youths.

**Methods:**

Nine focus group discussions and eight semi-structured interviews were conducted with 65 individuals aged 15-35 in Singapore. To ensure the comprehensive representation of youths’ perspectives, participants from diverse ethnicities (mainly Chinese, Malay, and Indian), ages, and genders were included using purposive sampling. The data was analysed using content analysis through both inductive and deductive approaches.

**Results:**

Four main themes were identified from the data. First, technology and platform: youths stressed the importance of a credible and government-endorsed service provider to deliver a comprehensive and trustworthy experience facilitated by qualified professionals. Second, functionality: they wanted credible affiliations to be displayed prominently on the home page and various tools such as calls, chats, moderated forums, profiles of healthcare professionals, and educational resources. Confidentiality, anonymity, and privacy were also highlighted as necessary. Third, user interface: youths preferred an intuitive and age-tailored interface to ensure a seamless and user-friendly experience, with organised content, appealing aesthetics, and engaging elements on video call sessions. Fourth, usability: they emphasised the need for an affordable and widely compatible operating system to promote accessibility of services.

**Conclusions:**

Virtual mental health services, with their great potential, can expand and effectively meet the needs of youths. By prioritizing credible platforms, comprehensive functionality, confidentiality, an intuitive interface, and broad accessibility, we can enhance help-seeking among youths and create a more effective support system.

**Disclosure of Interest:**

None Declared

